# Evaluation of plasma neurofilament light chain and glial fibrillary acidic protein in myasthenia gravis: A controlled cohort study

**DOI:** 10.1371/journal.pone.0352017

**Published:** 2026-07-07

**Authors:** Arta Grosmane-Bataraga, Evita Saluvēra, Marija Roddate, Vladimirs Krutovs, Kaj Blennow, Henrik Zetterberg, Maksims Zolovs, Nataļja Kurjāne, Viktorija Ķēniņa

**Affiliations:** 1 Rīga Stradiņš University, Department of Doctoral Studies, Riga, Latvia; 2 Pauls Stradiņš Clinical University Hospital, Department of Neurology, Riga, Latvia; 3 Rīga Stradiņš University, Faculty of Medicine, Riga, Latvia; 4 Department of Psychiatry and Neurochemistry, Institute of Neuroscience and Physiology, the Sahlgrenska Academy at the University of Gothenburg, Mölndal, Sweden; 5 Clinical Neurochemistry Laboratory, Sahlgrenska University Hospital, Mölndal, Sweden; 6 Department of Neurodegenerative Disease, UCL Institute of Neurology, London, United Kingdom; 7 UK Dementia Research Institute at UCL, London, United Kingdom; 8 Hong Kong Center for Neurodegenerative Diseases, InnoHK, Hong Kong, China; 9 Wisconsin Alzheimer’s Disease Research Center, University of Wisconsin School of Medicine and Public Health, University of Wisconsin-Madison, Madison, Wisconsin, United States of America; 10 Centre for Brain Research, Indian Institute of Science, Bangalore, India; 11 Daugavpils University, Institute of Life Sciences and Technology, Daugavpils, Latvia; 12 Riga Stradiņš University, Statistics Laboratory, Riga, Latvia; 13 Rīga Stradiņš University, Scientific Laboratory of Molecular Genetics, Riga, Latvia; 14 Pauls Stradiņš Clinical University Hospital, Centre of Clinical Immunology and Alleergology, Riga, Latvia; 15 Pauls Stradiņš Clinical University Hospital, Centre of Rare Neurological Diseases, Riga, Latvia; Cleveland Clinic Abu Dhabi, UNITED ARAB EMIRATES

## Abstract

**Aims:**

To evaluate plasma neurofilament light chain (NfL) and glial fibrillary acidic protein (GFAP) as candidate biomarkers in myasthenia gravis (MG).

**Methods:**

Ninety MG patients and 40 healthy controls were recruited. Disease severity was assessed by the Myasthenia Gravis Foundation of America (MGFA) classification, Myasthenia Gravis Composite (MGC) score, and Myasthenia Gravis Activities of Daily Living (MG-ADL) scale. Plasma NfL and GFAP were quantified using Single Molecule Array (Simoa) assays.

**Results:**

NfL and GFAP plasma concentration did not differ between MG and controls (*p* > 0.05). Neither biomarker correlated with MG-ADL or MGC, and no differences were observed across MGFA classes (p > 0.05). Biomarker levels were unrelated to myasthenic crisis history or treatment exposure.

**Conclusion:**

Plasma NfL and GFAP, although informative in other neuroimmunological and neurodegenerative conditions, do not distinguish MG from healthy controls and show no association with disease severity. This study adds to the emerging literature on NfL in MG and represents one of the larger controlled analyses incorporating both NfL and GFAP biomarkers in this disease. The findings argue against adopting NfL or GFAP for MG monitoring and highlight the need for MG-specific biomarker strategies.

## Introduction

Biomarkers play a pivotal role in neuroimmunology by providing objective measures of disease activity, prognosis, and treatment response. In conditions such as multiple sclerosis and neuromyelitis optica spectrum disorders, fluid biomarkers including neurofilament light chain (NfL) and glial fibrillary acidic protein (GFAP) have gained increasing attention as tools for monitoring neuroaxonal injury and astrocytic activation. In contrast, the biomarker landscape in myasthenia gravis (MG) remains poorly defined.

MG is a chronic autoimmune disorder targeting the neuromuscular junction, clinically characterised by fluctuating muscle weakness and fatigability [1]. The disease is mediated by pathogenic autoantibodies, most commonly against the acetylcholine receptor (AChR), muscle-specific kinase (MuSK), or lipoprotein receptor-related protein 4 (LRP4). Although antibody testing is invaluable for diagnosis, antibody titers do not consistently correlate with disease severity or prognosis [2]. Thus, there is an unmet need for reliable biomarkers that can predict disease generalization, monitor disease activity, and serve as outcome measures in clinical trials [3].

Recent studies have explored circulating microRNAs and serum protein signatures as potential MG biomarkers, but none have yet translated into clinical practice [4,5]. Neurofilaments (Nfs) are structural proteins that constitute a part of the axonal cytoskeleton. Nfs comprise the following subunits: α-internexin and neurofilament heavy, median and light (NfL) proteins [6]. Low levels of NfL proteins are continuously released from axons and tend to rise with age [7]. As a response to axonal damage in the central nervous system (CNS), the release of NfL sharply increases due to inflammation, neurodegeneration, trauma, and vascular injury [8]. Elevated NfL levels are well established in neurodegenerative and demyelinating CNS diseases, and more recently, in peripheral neuropathies. Similarly, some studies have detected higher plasma NfL levels in patient groups than in healthy controls outside the CNS, including in Charcot-Marie-Tooth disease and MG; however, correlations with disease severity have rarely been observed [9,10]. This biomarker profile is particularly well characterised in amyotrophic lateral sclerosis (ALS), another disorder affecting the motor unit. Serum NfL levels identify over 80% of patients with ALS and predict survival, outperforming both GFAP and pTau181. In contrast, GFAP did not show a significant pre-diagnostic association with incident ALS, remaining flat in the years preceding clinical onset, suggesting a more limited role in this condition. Given that ALS and MG both target the neuromuscular system, these findings provide a relevant framework for evaluating NfL and GFAP as candidate biomarkers in MG [11,12]. Preliminary reports in MG have suggested modest elevations, but findings have been inconsistent, and their clinical relevance remains uncertain.

Glial fibrillary acidic protein is a major component of the intermediate filament of the astrocyte cytoskeleton, and increased GFAP levels are thought to relate to reactive astrocytosis [13]. GFAP is expressed in both the CNS and peripheral nervous system (PNS) in non-myelinating immature Schwann cells and satellite cells of the dorsal root ganglia [14]. However, it is also likely that CNS and PNS GFAP are not identical since a GFAP monoclonal antibody has been found that selectively recognises GFAP in astrocytes but not in PNS cells [15]. Studies have shown that GFAP is also expressed in immature Schwann cells during the development and repair of Schwann cells after nerve injury [16]. However, to date, GFAP has not been systematically studied in MG.

The present study, therefore, aimed (1) to measure plasma concentrations of NfL and GFAP in patients with MG compared to healthy controls, and (2) to assess their potential associations with clinical severity, thereby evaluating their utility as candidate biomarkers for hypothesised diagnostic, prognostic and monitoring relevance in MG.

## Methods

### Participants

Patients were recruited between 1st of January and December 20th, 2023, during outpatient visits or hospital stays in a single centre setting. Inclusion criteria were: age ≥ 18 years, a confirmed diagnosis of MG, and provision of written informed consent. MG diagnosis was confirmed by a board-certified neurologist based on clinical presentation, serological antibody testing, and neurophysiological studies. Exclusion criteria included concomitant neurodegenerative disease, polyneuropathy, or malignancy.

The control group consisted of 40 healthy individuals without autoimmune disease or other relevant comorbidities.

### Clinical evaluation

Certified neurologists assessed the patients. Each patient’s demographic data, clinical information, and antibody status were collected during the visits.

Disease severity was assessed by the Myasthenia Gravis Foundation of America (MGFA) classification and MGFA post-intervention status (MGFA-PIS) classification [17], as well as by the Myasthenia Gravis Composite (MGC) score [18] and Myasthenia Gravis Activities of Daily Living (MG-ADL) scale [19].

### Blood sampling and measurement of plasma NfL and GFAP concentrations

Certified nursing staff acquired the patients’ blood samples during the visit or hospital stay. Blood sampling and subsequent storage procedures adhered to standard operating protocols. Blood samples were collected in tubes containing EDTA and processed within an hour after collection.

Samples were centrifuged at 20 °C and 3500 rpm for 10 minutes. The plasma was then carefully removed and stored at −20 °C. While maintaining the temperature, all samples were sent to plasma NfL and GFAP analysis. Plasma NfL and GFAP concentrations were measured using commercially available Single molecule array (Simoa) assays (Quanterix, Billerica, MA). All measurements were performed in one round of experiments using one batch of reagents by board-certified laboratory technicians blinded to the clinical data. Intra-assay coefficients of variation were below 10%.

### Statistical analysis

The primary objective was to determine whether plasma NfL and GFAP concentrations differ between MG patients and controls, and whether they associate with disease severity. Two prespecified hypotheses were tested: (i) plasma NfL and GFAP levels are elevated in MG compared with controls after adjusting for age and sex, and (ii) higher candidate marker concentrations are associated with greater disease severity (MGFA class, MGC score, MG-ADL). Secondary analyses evaluated associations with history of myasthenic crisis, exposure to rescue therapies [intravenous immunoglobulin (IVIg), plasma exchange (PEX)] at any time during the disease course, and current maintenance treatment regimens at the time of the blood sampling. The analyses were designed to evaluate associations between biomarker concentrations and treatment exposure categories/current immunosuppressive regimens rather than dose-dependent treatment effects. Therefore, medication dosing was not incorporated into the statistical models.

Continuous variables were summarized as mean (SD) or median (IQR), and categorical variables as frequencies (%). Between-group comparisons were performed using the Kruskal–Wallis test for continuous variables and χ² or Fisher’s exact test for categorical variables. Correlations were assessed using Spearman’s rank test. Multivariable linear and logistic regression models were used to assess associations between NfL and GFAP and clinical measures, adjusting for age and sex.

Statistical analyses were performed using *Jamovi* (v2.5). Figures were generated in *IBM SPSS Statistics*. Statistical significance was set at *p* < 0.05.

### Ethics

The study was conducted in accordance with the Declaration of Helsinki. Written informed consent was obtained from all participants. The local research ethics committee approved the protocol.

## Results

### Patient demographics

A total of 90 patients with MG (37 males [41%], 53 females [59%]) and 40 healthy controls were included. The mean age of the MG cohort was 57.2 years (SD ± 14.7, 95% CI 54.1–60.3); males were slightly older than females (59.6 ± 12.0 vs 54.7 ± 16.6 years). Sex distribution was similar between groups (χ² = 0.075, *p* = 0.784). Mean age differed between patients and controls but did not reach statistical significance (*t* = 1.734, *p* = 0.087). Detailed group characteristics are provided in Table 1.

**Table 1:** Characteristics of the patient and control groups.

	Total MG patient group	Control group
Patients (n, %)	n=90 (100%)	n=40 (100%)
Sex, male/female (n, %)	37 (41%)/53 (59%)	11 (28%)/29 (72%)
Mean age in years (SD)	57.2 (±14.7)	41.5 (±11.1)
Median disease duration (IQR), months	100.0 (±121.6)	N/A
Antibody status (n, %)		
• Anti-AChR	79 (88%)	N/A
• Anti-MuSK	4 (4%)	
• Seronegative	7 (8%)	
MGFA status (n, %)		
• I	14 (16%)	N/A
• IIa	15 (17%)	
• IIb	18 (20%)	
• IIIa	2 (2%)	
• IIIb	12 (13%)	
• IVb	2 (2%)	
• Total or pharmacological remission	27 (30%)	
Treatment regimen (n, %)		
• AchE-I + CS + NSIS	40 (44%	N/A
• CS + NSIS	3 (3%)	
• AchE-I + NSIS	10 (11%)	
• AchE-I + CS	12 (13%)	
• AchE-I	14 (16%)	
• CS	3 (3%)	
• No therapy	8 (9%)	
Additional therapy (n, %)		
• MCAB (Rituximab)	1 (1%)	
• IvIg	13 (14%)	
• PLEX	26 (29%)	

Table showing the MG and control patient demographics and clinical data. Abbreviations: AchE-I: acetylcholinesterase inhibitors; AChR: acetylcholine receptor; CS – corticosteroids; IQR: interquartile range; MG: myasthenia gravis; IvIg: intravenous immunoglobulin; MCAB: monoclonal antibodies; MGFA – Myasthenia Gravis Foundation of America, MuSK – muscle-specific kinase, N/A: not applicable; NSIS: non-steroid immunosuppressants; PLEX: plasma exchange; SD: standard deviation.

### Associations of NfL and GFAP with age and sex

Age showed a strong positive association with both NfL and GFAP. Each additional year of age predicted higher plasma NfL concentrations (estimate 0.33, 95% CI 0.17–0.49, *p* < 0.001) and GFAP concentrations (estimate 2.70, 95% CI 1.17–4.23, *p* < 0.001). These relationships remained significant after adjusting for sex, disease duration, MGFA class, and treatment status. No independent effect of sex was observed.

### Group comparisons (MG vs controls)

Plasma concentrations of NfL and GFAP did not differ significantly between MG patients and controls after adjustment for age and sex. The association between MG status and the studied outcome appeared weak, with wide confidence intervals suggesting substantial uncertainty and insufficient evidence for a definitive conclusion. Median concentrations for each group are shown in Table 2.

**Table 2:** Plasma NfL and GFAP concentration comparisons between the patient group and the control group.

	MG group	Control group
Median NfL, pg/mL (IQR)	9.8 (9.3)	6.1 (4.0)
Median GFAP, pg/mL (IQR)	122.5 (99.6)	87.5 (48.5)
Median MGC score (IQR)	4.0 (8.0)	N/A
Median MG-ADL scale (IQR)	3.0 (6.0)	N/A
NfL correlation with age, *p*	0.714, *p* < 0.001	0.481, *p* = 0.002
GFAP correlation with age, *p*	0.618, *p* < 0.001	0.303, *p* = 0.57
NfL correlation with disease duration, *p*	−0.165, *p* = 0.125	N/A
GFAP correlation with disease duration, *p*	−0.080, *p* = 0.457	N/A
NfL correlation with MGC score, *p*	−0.080, *p* = 0.458	N/A
NfL correlation with MG-ADL scale, *p*	−0.052, *p* = 0.629	N/A
GFAP correlation with MGC score, *p*	−0.058, *p* = 0.593	N/A
GFAP correlation with MG-ADL scale, *p*	−0.079, *p* = 0.463	N/A

Table showing the median plasma neurofilament light chain (NfL) and glial fibrillary acidic protein (GFAP) concentration in the myasthenia gravis and control groups, correlation with age and median results of the scales. Abbreviations: IQR: interquartile range; MG: myasthenia gravis; MGC score: Myasthenia Gravis Composite score; MG-ADL scale: Myasthenia Gravis Activities of Daily Living scale; N/A: not applicable; GFAP: plasma glial fibrillary acidic protein; NfL: plasma neurofilament; SD: standard deviation.

### Associations with disease severity

**Fig 1:**
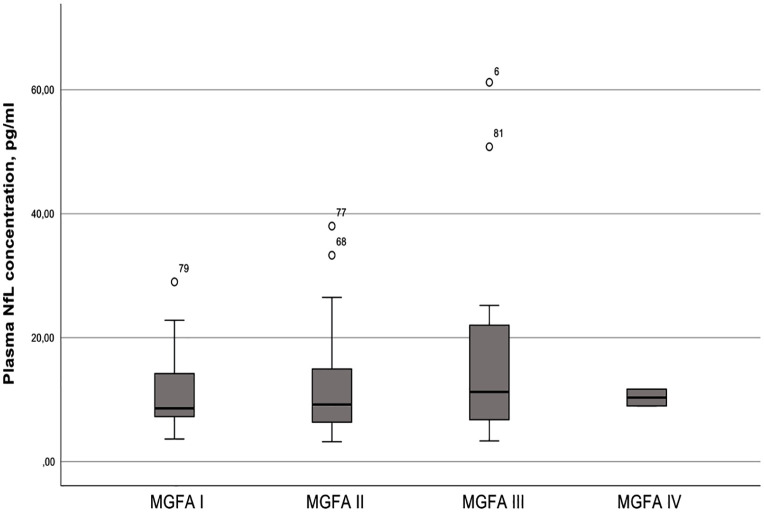
Plasma neurofilament light (NfL) concentration across the Myasthenia Gravis Foundation of America (MGFA) severity groups. A boxplot graph showing NfL concentration (pg/ml) across Myasthenia Gravis Foundation of America severity groups. No significant difference in NfL concentration across MGFA cgroups was found (p = 0.662).

**Fig 2:**
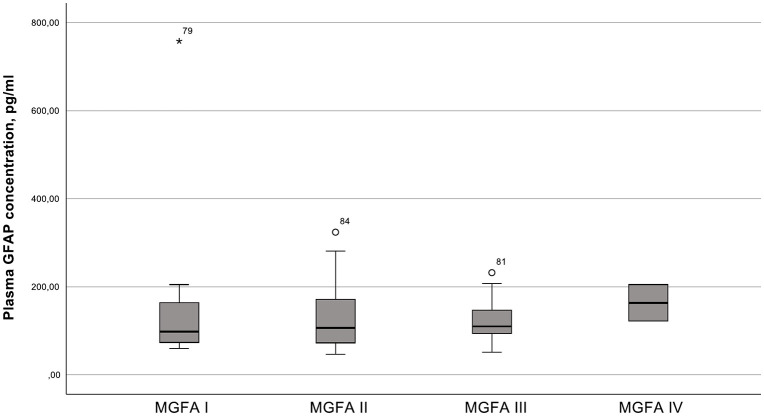
Plasma glial fibrillary acidic protein (GFAP) across the MG severity groups. A boxplot graph showing NfL concentration (pg/ml) across Myasthenia Gravis Foundation of America severity groups. There was no significant difference in GFAP concentration across MGFA groups (p = 0.331).

Within the MG cohort, plasma NfL and GFAP concentrations were not associated with clinical severity. Neither candidate marker correlated with MGFA class (Fig 1 and Fig 2), MGC score, nor MG-ADL scale (*p* > 0.05 for all comparisons).

### Clinical outcomes and treatment exposures

No significant associations were identified between candidate marker concentrations and history of myasthenic crisis, exposure to rescue therapies (IVIg, PEX), or current treatment regimen. Multivariable logistic and multinomial regression analyses yielded non-significant results throughout (*p* > 0.05).

## Discussion

This study found no significant differences in plasma concentrations of neurofilament light chain or glial fibrillary acidic protein between patients with myasthenia gravis and healthy controls. Neither candidate marker correlated with disease severity as assessed by MGFA class, MGC score, or MG-ADL, nor with history of crisis or treatment exposure. These findings suggest that, unlike in other neuroimmunological conditions, NfL and GFAP are not suitable plasma biomarkers for MG.

In contrast to earlier reports suggesting elevated NfL in MG subgroups, our age-adjusted analyses did not demonstrate a disease-specific increase. Prior studies included older patients compared with controls, whereas our cohort was more closely age-matched. Because NfL strongly correlates with age, previously reported elevations may have been driven by demographic differences rather than MG pathology. This emphasizes the need for age adjustment when interpreting NfL levels and indicates that NfL does not capture MG-specific pathophysiological changes.

Elevated NfL levels have been linked to reduced muscle performance and atrophy, features seen in MG patients who are often physically inactive [20,21]. Histopathological studies suggest that muscle fibre atrophy may occur secondary to denervation of the motor endplate, potentially contributing to neurofilament release [22–24]. Reports of increased neurofilament heavy chain in ocular MG support this mechanism, but our findings provide no evidence of such changes in plasma NfL [25]. Our findings suggest that neuromuscular junction pathology is not associated with detectable neurofilament release. While larger studies integrating histopathology, neurophysiology, and biomarker analyses could provide additional confirmation, major changes in the observed relationship are unlikely if well-defined study populations are used.

GFAP, in contrast, has not previously been studied in MG. While primarily expressed in astrocytes, GFAP is also present in terminal Schwann cells at the neuromuscular junction [26,27]. In CNS disorders such as traumatic brain injury, multiple sclerosis, and Alzheimer’s disease, GFAP serves as a robust marker of astrocytic injury. Based on histopathological evidence of secondary motor endplate denervation, we hypothesized that GFAP might also be increased in MG. However, plasma concentrations did not differ between patients and controls. GFAP correlated with age only in the whole MG cohort, consistent with prior reports that increases become more evident in older populations [28–30]. Given that our age-adjusted MG and control groups were relatively young, it is possible that GFAP changes in MG might only be detectable in older cohorts [30].

The absence of biomarker utility for NfL and GFAP in MG highlights important differences between MG and other neuroimmunological disorders, such as multiple sclerosis (MS) and neuromyelitis optica spectrum disorders (NMOSD). In MS and NMOSD, NfL reflects axonal injury and GFAP indicates astrocytic damage, both of which are central to disease progression. By contrast, MG primarily affects the neuromuscular junction, where postsynaptic receptor dysfunction rather than widespread axonal or astrocytic injury drives clinical manifestations. Although secondary changes such as denervation and muscle atrophy may occur, they appear insufficient to produce robust or consistent increases in circulating NfL or GFAP [31]. These differences underscore that biomarkers validated in CNS-driven disorders cannot be assumed to translate to MG, where pathology is localised and immune-mediated in a distinct manner. Our findings therefore reinforce the necessity of developing disease-specific biomarker strategies for MG, focusing on mechanisms directly linked to its pathophysiology, such as autoantibody profiles, immune cell phenotyping, cytokine signatures, and circulating microRNAs.

This study has several limitations that should be acknowledged. The control group was relatively small and younger than the whole MG cohort, necessitating age adjustment. In addition, seronegative and MuSK-positive subgroups were underrepresented, precluding meaningful comparisons with AChR-positive patients. Finally, the distribution across MGFA severity classes was imbalanced, with small numbers in higher severity groups, requiring class combinations for analysis. These factors may have reduced the power to detect subtle subgroup differences.

In conclusion, this study demonstrates that plasma neurofilament light chain and glial fibrillary acidic protein, despite their established value as biomarkers in other neuroimmunological and neurodegenerative disorders, do not differentiate patients with myasthenia gravis from healthy controls and show no association with disease severity or treatment exposure. As the first systematic evaluation of GFAP in MG and one of the largest controlled studies of NfL in MG, our findings highlight that biomarkers validated in central nervous system diseases cannot be directly applied to MG. Future research should therefore prioritize the development of MG-specific biomarker strategies, focusing on immunological and molecular mechanisms more closely aligned with the pathophysiology of this rare disorder.

## Supporting information

S1 Dataset This is the dataset underlying the findings of this study. (XLSX)
