# Supplementary material for: Evaluation of plasma neurofilament light chain and glial fibrillary acidic protein in myasthenia gravis: A controlled cohort study
Source: PLoS One. 2026 Jul 7;21(7):e0352017. doi: 10.1371/journal.pone.0352017 (PMC13340798; doi:10.1371/journal.pone.0352017)
Supplement: Attachment — Submitted filename: point-to-point response.docx [file pone.0352017.s003.docx]

**Response to the Editor and Reviewer**

We thank the Editor and the Reviewers for their careful and thorough evaluation of our manuscript and for their constructive comments. We have revised the manuscript accordingly and believe these changes have improved the paper’s clarity and quality. Below, we provide a detailed, point-by-point response to each comment. All revisions have been incorporated into the manuscript and highlighted in the revised version.

We hope that the revisions and responses adequately address the reviewers’ comments and improve the manuscript. We appreciate the opportunity to revise our work and look forward to your further consideration.

**Editorial comments**

**1. Please ensure that your manuscript meets PLOS ONE's style requirements, including those for file naming. The PLOS ONE style templates can be found at**
[**https://journals.plos.org/plosone/s/file?id=wjVg/PLOSOne_formatting_sample_main_body.pdf**](https://journals.plos.org/plosone/s/file?id=wjVg/PLOSOne_formatting_sample_main_body.pdf) **and** [**https://journals.plos.org/plosone/s/file?id=ba62/PLOSOne_formatting_sample_title_authors_affiliations.pdf**](https://journals.plos.org/plosone/s/file?id=ba62/PLOSOne_formatting_sample_title_authors_affiliations.pdf)

**Response.** We thank the editors for this guidance. The manuscript has been revised to comply with PLOS ONE style requirements, including formatting, structure, and file naming, using the provided templates.

**2. Your ethics statement should only appear in the Methods section of your manuscript. If your ethics statement is written in any section besides the Methods, please delete it from any other section.**

**Response.** We confirm that the ethics statement appears only in the Methods section of the revised manuscript (page 8, lines 157-160).

3**. We note that you have indicated that there are restrictions to data sharing for this study. For studies involving human research participant data or other sensitive data, we encourage authors to share de-identified or anonymized data. However, when data cannot be publicly shared for ethical reasons, we allow authors to make their data sets available upon request. For information on unacceptable data access restrictions, please see http://journals.plos.org/plosone/s/data-availability#loc-unacceptable-data-access-restrictions.
Before we proceed with your manuscript, please address the following prompts:
a) If there are ethical or legal restrictions on sharing a de-identified data set, please explain them in detail (e.g., data contain potentially identifying or sensitive patient information, data are owned by a third-party organization, etc.) and who has imposed them (e.g., a Research Ethics Committee or Institutional Review Board, etc.). Please also provide contact information for a data access committee, ethics committee, or other institutional body to which data requests may be sent.
b) If there are no restrictions, please upload the minimal anonymized data set necessary to replicate your study findings to a stable, public repository and provide us with the relevant URLs, DOIs, or accession numbers. Please see http://www.bmj.com/content/340/bmj.c181.long for guidelines on how to de-identify and prepare clinical data for publication. For a list of recommended repositories, please see https://journals.plos.org/plosone/s/recommended-repositories. You also have the option of uploading the data as Supporting Information files, but we would recommend depositing data directly to a data repository if possible.
Please update your Data Availability statement in the submission form accordingly.**

**Response.** We agree with the importance of ensuring transparency and reproducibility. In the original submission, the wording regarding data availability did not align with PLOS policy and may have caused confusion. We have now revised this to ensure compliance. The dataset underlying the findings of this study will be provided as Supplementary Information accompanying the revised manuscript (Supplementary information, file name - S1_Dataset).

**4. In the online submission form, you indicated that anonymised data that support the findings of this study are available from the corresponding author upon reasonable request.**
**All PLOS journals now require all data underlying the findings described in their manuscript to be freely available to other researchers, either 1. In a public repository, 2. Within the manuscript itself, or 3. Uploaded as supplementary information.
This policy applies to all data except where public deposition would breach compliance with the protocol approved by your research ethics board. If your data cannot be made publicly available for ethical or legal reasons (e.g., public availability would compromise patient privacy), please explain your reasons on resubmission and your exemption request will be escalated for approval.**

**Response.** We thank the editor for highlighting this important issue. In the original submission, the Data Availability Statement was not fully aligned with PLOS ONE policy and may have caused confusion. We have now revised this to ensure full compliance. The minimal anonymised dataset required to replicate the study’s findings is provided in the Supporting Information accompanying the manuscript (S1_Dataset). The dataset has been fully de-identified and does not contain any personally identifiable information. Data sharing complies with ethical approval and applicable data protection regulations.

**5.** [**Please include captions for your Supporting Information files at the end of your manuscript, and update any in-text citations to match accordingly. Please see our Supporting Information guidelines for more information: http://journals.plos.org/plosone/s/supporting-information.**](http://journals.plos.org/plosone/s/supporting-information.)

**Response.** We have added Supporting Information at the end of the manuscript (page 21, line 416), which includes a caption for the supplementary file (the dataset underlying the study's findings).

**6. We note that there is identifying data in the Supporting Information file <Ethics_Committee_Approval_English.docx>. Due to the inclusion of these potentially identifying data, we have removed this file from your file inventory. Prior to sharing human research participant data, authors should consult with an ethics committee to ensure data are shared in accordance with participant consent and all applicable local laws.**

**Response.** The file “Ethics_Committee_Approval_English.docx” contains the Ethics Committee's official decision, including the names of the committee members as part of the formal approval document. These names refer to institutional representatives acting in their professional capacity and do not constitute research participant data. Nevertheless, we fully acknowledge the importance of safeguarding potentially identifying information. In accordance with your guidance, we have removed the file from the Supporting Information. If required, we are happy to provide a redacted version of the document or submit the approval confidentially for editorial review.

**7. If the reviewer comments include a recommendation to cite specific previously published works, please review and evaluate these publications to determine whether they are relevant and should be cited. There is no requirement to cite these works unless the editor has indicated otherwise.**

**Reviewer 1.**

**1. Why are there restrictions on data availabiity? Later the authors state that the data can be requested. If the former was a mistake, please correct it.**

**Response.** We thank the reviewer for highlighting this point. As stated above in the response to editorial comments, we agree with the importance of ensuring transparency and reproducibility. In the original submission, the wording regarding data availability did not align with PLOS policy and may have caused confusion. We have now revised this to ensure compliance.

The dataset underlying the findings of this study will be provided as Supplementary Information accompanying the revised manuscript. We confirm that the data have been fully anonymized and do not contain any identifiable personal information, and their sharing is in accordance with applicable ethical and data protection regulations.

**2. I would recommend refraining from using the word "limited" in the title. As they point out in the discussion and conclusion sections, they were unable to find statistically significant associations between these biomarkers, MG presence and various different characteristics. Therefore this paper actually supports lack of utility. Furthermore, generally phrasing the research question rather than making a statement in the title is preferable.**

**Response.** We agree with this important suggestion. The title has been revised to remove interpretative wording (“limited utility”) and instead reflect the research question in a neutral manner (page 1, lines 1-3).

**3. Biomarkers can be diagnostic, prognostic, predictive/risk-related, therapeutic/monitoring, or safety-related. Please define the hypothesized role of NFL and GFAP biomarkers in MG in the introduction section (which appears to be both diagnostic and prognostic based on the variables selected for studying). Additionally, these should not be referred to as biomarkers in the rest of the paper as it was not shown that they have such a role in MG.**

**Response.** We thank the reviewer for this important comment. We agree that the original wording could overstate the established role of NfL and GFAP in MG. We have revised the Introduction to clarify that NfL and GFAP were investigated as candidate biomarkers with hypothesised diagnostic and prognostic/monitoring relevance in MG (page 5, lines 99-100)

We also revised the wording throughout the manuscript to avoid implying that the utility of biomarkers has been demonstrated in MG, replacing the word “biomarker” with “NfL and GFAP” or “candidate markers” where appropriate.

**4. In the introduction, I would recommend mentioning the role of NFL (as well as GFAP) in ALS given its relevance as both ALS and MG are neuromuscular disorders.**

**Response.** We thank the reviewer for this helpful suggestion. We have revised the Introduction to include the established role of NfL and GFAP in ALS, highlighting their relevance in neuromuscular disorders and further supporting the rationale for investigating these biomarkers in MG (page 5, lines 79-86).

**5. Line 79, MG is not a peripheral neuropathy. Please rephrase this sentence.
Response.** We agree with this note. The sentence has been rephrased so that it does not include incorrect terminology (pages 4-5, lines 76-78).

**6. In the methods section, the authors should better define the variables included in this study. For example, does exposure to rescue therapies refer to any history of rescue therapy, or recently? These would have different implications. Similarly, does treatment regimen refer to current therapies? How are prior therapies factored into the analyses (given patients with prior therapy escalation likely have more severe disease)? Are different doses of treatments taken into consideration during analyses?**

**Response.** We thank the reviewer for highlighting the need for clearer definitions of treatment-related variables.

Specifically, “exposure to rescue therapies” referred to any documented prior use of intravenous immunoglobulin (IVIg) or plasma exchange (PEX) at any time during the disease course, rather than only recent treatment exposure. “Treatment regimen” referred to the patient’s current maintenance therapy at the time of blood sampling. This has now been described in the Methods section (page 7, lines 142-144).

We agree that prior treatment escalation may reflect more severe disease. To address this, the history of rescue therapy exposure and current treatment category were analysed separately from clinical severity measures and were interpreted as exploratory variables rather than independent markers of disease activity.

Regarding treatment dosages, the primary purpose of treatment-related analyses was to explore whether patients receiving immunosuppressive therapies differed in NfL/GFAP concentrations compared with other treatment groups, rather than to evaluate dose-response relationships. This has been clarified in the Methods section (page 7, lines 144-147).

**7. Lines 177-178, it is not clear what is meant in this sentence, please rephrase.**

**Response.** We thank the reviewer for pointing this out. The sentence has been rephrased to clarify that the association with MG and NfL and/or GFAP was weak and that the wide confidence intervals provided insufficient evidence for conclusions (page 10, lines 186-188).

**8. There are stylistic and scientific issues with the discussion section.**

**a) Lines 237-289, this sentence was already mentioned in the introduction, please do not repeat here. The authors should rather explain they chose to study these specific proteins as potential biomarkers in MG - what is the scientific rationale of potential relevance in MG? This is clarified to some extent in the following paragraphs, however this explanation should be provided early on in the paper.
Response.** We agree with the remark and have removed the repeated sentence from the discussion section.

**b) Lines 246-247: This sentence is rather redundant and if intended to keep, should be moved to the prior paragraph.**

**Response.** We agree with the remark and have removed the sentence from the discussion section.

**c) Lines 252-255: This is a broad and generic sentence without justification provided - what is testing in a larger study indicated if the results from this study are negative?**

**Response.** We thank the reviewer for pointing this out and have rephrased the sentence to better reflect the interpretation of our findings. The revised text now emphasises that our study did not demonstrate a meaningful association between neuromuscular junction pathology and detectable neurofilament release, and that while larger studies may provide additional confirmation, substantially different results are unlikely if appropriately selected and well-characterised study groups are used (page 13, lines 259-263).

**9. In both the conclusion paragraph and abstract section, authors claim that this is the first study to systematically assess the role of NFL (and GFAP) in MG. This is not true, especially with regards to NFL. There are other studies which looked at NFL in MG: PMID: 40988756, PMID: 36712429. Please change this sentence to focus on novelty rather than a claim of primacy.**

**Response.** We thank the reviewer for this important comment. We agree that the original wording overstated the novelty of the study, particularly with respect to NfL, as previous studies have already investigated NfL in MG (PMID: 40988756, PMID: 36712429). We have therefore revised the sentence in both the Abstract (page 3, line 48) and Discussion (page 15, lines 299-300) sections to avoid claims of primacy.

**Other minor correctios:**

- The last name of the first author has been changed to Arta Grosmane-Bataraga.
- One sentence was modified and another removed (page 14, lines 281-283 in the original manuscript) from the Discussion section, as they described limitations of an earlier statistical design that is not relevant to either the original or the revised manuscript.
